# Sleep quality and health-related quality of life among long-term survivors of (non-) Hodgkin lymphoma in Germany

**DOI:** 10.1371/journal.pone.0187673

**Published:** 2017-11-06

**Authors:** Friederike Hammersen, Philip Lewin, Judith Gebauer, Ilonka Kreitschmann-Andermahr, Georg Brabant, Alexander Katalinic, Annika Waldmann

**Affiliations:** 1 Institute for Social Medicine and Epidemiology, University of Luebeck, Luebeck, Germany; 2 Experimental and Clinical Endocrinology Department of Internal Medicine I, University Hospital of Schleswig-Holstein, Campus Luebeck, Luebeck, Germany; 3 Department of Neurosurgery, University Hospital Essen, University of Duisburg-Essen, Essen, Germany; 4 Institute for Cancer Epidemiology e.V., University of Luebeck, Luebeck, Germany; 5 Hamburg Cancer Registry, Hamburg, Germany; Universita degli Studi di Roma La Sapienza, ITALY

## Abstract

This study investigated sleep quality and health-related quality of life (HRQOL) among long-term survivors of Hodgkin (HL) and non-Hodgkin lymphoma (NHL). The aim was to explore the impact of personal and health-related factors on sleep quality as well as associations between sleep quality and HRQOL. For the postal survey, participants with a minimum age of 18 years initially treated between 1998 and 2008 were recruited via the population-based cancer registry in Schleswig-Holstein, Northern Germany. Questionnaires included amongst others the Pittsburg Sleep Quality Index (PSQI) and the 36-Item Short Form Health Survey (SF-36v1). Descriptive and comparative statistics were performed. Additionally, a regression analysis was conducted to identify predictors of sleep quality. In total, we recruited 515 participants (398 NHL, 117 HL) with a mean age of 63.1 years. Approximately half of the survivors were classified as good sleepers. HRQOL scores differed between good and poor sleepers with lower scores in poor sleepers. In a prediction model, self-reported depression, exhaustion, higher age, inability to work, endocrinological disorders and female gender classified as predictors of sleep quality. This study highlights the impact of sleep quality on HRQOL in long-term survivors of NHL and HL. Thus, sleep quality should be routinely assessed during follow-up of cancer survivors with special attention to patients with potential risk factors.

## Introduction

In Germany as in other industrialized countries, cancer incidences increased over the last decades, while mortality declined [1]. This is largely due to improvements in early detection and treatment of various types of cancer [2, 3]. Consequently, high survival rates are no longer the only treatment goal as survivors’ long-term quality of life and especially their health-related quality of life (HRQOL) attract increasing attention [4]. Besides its immediate relevance for cancer patients’ well-being, HRQOL was also found to be a prognostic factor for overall survival [5, 6]. Relevant factors known to be associated with HRQOL are sleep quality, insomnia and fatigue [7–11], whereby poor sleep quality often occurs as a side-effect of cancer [8, 12–16]. Evaluation of sleep quality in epidemiological studies is, however, affected by several factors including the applied instruments and their validity and reliability as well as the time of measurement, e.g. at diagnosis, during treatment or afterwards. Within these limitations, the overall prevalence of sleep disturbances in cancer patients is currently estimated to range between 26% and 59% [15, 17].

Studies concerning sleep and HRQOL, respectively, have previously most often been conducted on breast cancer patients [7, 10, 12, 14]. Fewer studies exist on Hodgkin (HL) and non-Hodgkin lymphoma (NHL) survivors. These entities are of special interest though as they constitute a continuously growing group of younger cancer survivors with a generally good long-term prognosis. Existing studies indicate significantly increased prevalence of fatigue in long-term survivors of HL and NHL when compared to the general population [16, 18, 19]. Fatigue may be considered a potential surrogate marker for decreased sleep quality. Additionally, lymphoma survivors also reported a worse HRQOL [8, 16, 20, 21]. This is related to HL patients’ impaired working abilities as they more often work part-time or are unemployed due to health-related problems [21]. The causes of these cancer- and/or therapy-related sleep disorders along with the worse HRQOL are currently unclear. Clinical factors (e.g. diagnosis, stage of cancer, therapy-associated endocrine late effects), physical complaints (e.g. pain, nausea), psychological and environmental factors as well as medications and their side effects may also be discussed [8, 17, 22].

The aim of the present study was to demonstrate long-term effects of cancer in survivors of NHL and HL with special attention to sleep quality and HRQOL, as regular follow-up of cancer patients often ends after 5 years of recurrence-free survival. The approach of Buysse [23] was applied including quantitative aspects of sleep quality, like sleep duration and sleep latency, as well as more subjective aspects, like sleep depth and restfulness [23]. Additionally, we assessed possible socio-demographic, social as well as health-related predictors of sleep quality to identify potential underlying mechanisms. Furthermore, we focused on the interaction of sleep quality and HRQOL.

## Subjects and methods

### Recruitment of study participants

Potential study participants for the “Endocrine late effects after (non-)Hodgkin lymphoma-study” (ELLY) were identified via the population-based cancer registry in Schleswig-Holstein, the most Northern federal state in Germany. We included patients with a diagnosis of HL (ICD-10 C81) or NHL (ICD-10 C82, C83, C84, C85), who were diagnosed and initially treated between 1998 and 2008. Further inclusion criteria were a minimum age of 18 years at diagnosis and a given consent to be contacted for scientific purposes. Eligible patients (n = 942) were invited via mail to participate in the postal survey. Questionnaires included instruments covering subjective sleep quality and HRQOL. More details regarding the study and questionnaires have been described elsewhere [22].

### Measurement of sleep quality

Self-reported sleep quality was measured with the Pittsburgh Sleep Quality Index (PSQI) [23]. The instrument contains 19 items pertaining to the sleep of the preceding 4 weeks. It produces a global score, which consists of 7 component scores: sleep quality, sleep latency, sleep duration, habitual sleep efficiency, sleep disturbances, use of sleeping medications, and daytime dysfunction. The global score ranges from 0 to 21. Higher scores represent a worse sleep quality (also in the component scores). With the help of a cut-off value (≤ 5), study populations can be dichotomized into good and poor sleepers [23].

### Measurement of HRQOL, depression and health perception

HRQOL was assessed with the 36-Item Short Form Health Survey (SF-36v1), a reliable and validated generic self-rating instrument [24, 25]. The SF-36v1 is bi-dimensional with 8 subscales, which can be allocated onto two basic dimensions; the physical (PCS) and the mental health component summary score (MCS) [25]. In our study, we transformed the raw values into norm-based scores to allow for comparisons with the German population (mean = 50 ± SD 10) [24]. The transformed scales range from 0 to 100, have a mean of 50 and a SD of ±10. Higher scores indicate a better HRQOL. A score 1 SD below the mean (<40) then indicates a below-average HRQOL [24], which we decided to label as an impaired HRQOL as compared to a good HRQOL (50± SD 10). A score below 2 SD (<30) reflects a severely impaired HRQOL.

The World Health Organisation-5 (WHO-5) well-being index was used to screen for the presence of a clinically relevant depression [26]. It consists of 5 questions referring to the last two weeks, with a maximum of 5 points per question adding up to a sum score. We used the cut-off value of 7, as a sum score of less than 7 points is most likely to indicate a clinically relevant depression [27, 28].

Additionally, the survivors’ own health perception was assessed by two statements. The first one assessed whether they felt cured, experienced a recurrence, metastases or other secondary malignant diseases. For the analysis, we dichotomized the answers into an affirmative response to “I am cured” or not (labeled as health concept). The second statement, which was to be answered on a yes/no basis, assessed if, after their malignant disease, patients felt an increased lack of energy, tiredness and inefficiency.

### Statistics

The study population were described with absolute and relative frequencies, arithmetic means, standard deviations (SD), medians and ranges and, where appropriate, with 95% confidence intervals (CI), respectively. Proportions relate to the number of participants who answered the question. Regression analysis was conducted to investigate predictors of sleep quality in cancer survivors. In order to be able to include cancer entity as a predictor, the participants were treated as one group. The following predictors were included in the regression analysis: socio-demographic factors and tumor entity (variables: age, entity), social setting (marital and occupational status) and health-related factors (number of endocrinological co-morbidities, likely presence of clinically relevant depression, health concept, exhaustion). The selection of these variables was theory-driven. Age at survey was grouped into 4 categories, which were derived from the quartiles of the continuous variable. Endocrinological co-morbidities were categorized as present if the participants had ever been diagnosed with one or more of the following conditions: dyslipidemia, hyperthyroidism, hypothyroidism, nodes of the thyroid gland, struma diffusa, diabetes mellitus and osteoporosis, respectively. The results are indicated with the non-standardized regression coefficient β and the adjusted and non-adjusted R^2^, respectively. To investigate the influence of sleep quality on HRQOL, the means of the transformed, norm-based SF-36 subscales as well as of the component summary scores were compared between good and poor sleepers with the help of a t-test. The sleep classification was based on the recommended cut-off value of the PSQI (≤ 5) [23]. SPSS version 22 (IBM) was used to analyze the data. A p-value of <0.05 was regarded as significant.

### Ethics

The local ethics committee of the University of Luebeck reviewed and approved the study protocol (reference number 13–014; decision letter dated April 03^rd^, 2013). Informed consent to be contacted was obtained by all patients at the time of registration to the cancer registry. The questionnaire included written informed consent. All procedures performed in studies involving human participants were in accordance with the ethical standards of the institutional and/or national research committee and with the 1964 Helsinki declaration and its later amendments or comparable ethical standards.

## Results

### Study participants

Overall, 942 eligible persons were contacted, of whom 515 responded to the study (response rate 54.7%). There were no clinical relevant or statistical differences in baseline variables between responding and non-responding persons regarding clinical characteristics as documented in the cancer registry. Detailed descriptions of non-/responders can be found elsewhere [22].

The final study population included 222 women (43.1%) and 293 men (56.9%) (Table 1). Among these, 398 patients were diagnosed with NHL and 117 patients with HL. Only a minority (7.2%, n = 31) had suffered from other earlier cancer entities. At the time of the survey, the participants’ mean age was 63.1 years (±14.4) and approximately 9 years had passed since the cancer diagnosis. The great majority of survivors (83.4%, n = 403) had received chemotherapy, followed by radiotherapy (70.4%, n = 300). Most participants were married (67.6%). Survivors of NHL were older and more often retired than HL survivors, whereas more full-time working participants were found among the HL survivors (46.0%, n = 51). The proportion of premature pensioners and disabled persons was comparably low in both entities.

**Table 1:** Characteristics of the study population.

	HL n = 117	NHL n = 398	total n = 515
**sex**^a^: female	46 (39.3)	176 (44.2)	222 (43.1)
**age at diagnosis (years)**^a^			
mean ± SD	42.4 (15.5)	56.7 (12.5)	53.4 (14.5)
median (min–max)	41 (18–79)	59 (18–79)	56 (18–79)
**time since diagnosis (years)**^a^			
mean ± SD	9.7 (3.2)	9.0 (2.9)	9.2 (3.0)
median (min–max)	10.0 (4–15)	9.0 (4–15)	9.0 (4–15)
**age at survey (years)**^a^			
mean ± SD	52.4 (15.5)	66.2 (12.4)	63.1 (14.4)
median (min–max)	51.0 (24–88)	69.0 (25–88)	65.0(24–88)
**marital status**			
married	68 (60.7)	268 (69.6)	336 (67.6)
married, but living apart	0 (0.0)	6 (1.6)	6 (1.2)
divorced	11 (9.8)	34 (8.8)	45 (9.1)
widowed	7 (6.3)	50 (13.0)	57 (11.5)
single	26 (23.2)	27 (7.0)	53 (10.7)
**occupational status**			
retirement	28 (25.2)	221 (59.9)	249 (51.9)
premature pension / disability	11 (9.9)	44 (11.9)	55 (11.5)
illness / unable to work	3 (2.7)	6 (1.6)	9 (1.9)
fulltime	51 (46.0)	57 (15.5)	108 (22.5)
part-time	12 (10.8)	22 (6.0)	34 (7.1)
housework	2 (1.8)	13 (3.5)	15 (3.1)
unemployed	1 (0.9)	2 (0.5)	3 (0.6)
education / studies / reeducation	3 (2.7)	4 (1.1)	7 (1.5)
**currently working**			
yes	73 (64.6)	101 (27.1)	174 (35.8)
no	40 (35.4)	272 (72.9)	312 (64.2)
**surgery**^a^			
yes	57 (59.4)	171 (51.5)	228 (53.3)
no	39 (40.6)	161 (48.5)	200 (46.7)
missing	21 (-)	66 (-)	87 (-)
**chemotherapy**^a^			
yes	111 (97.4)	292 (79.1)	403 (83.4)
no	3 (2.6)	77 (20.9)	80 (16.6)
missing	3 (-)	29 (-)	32 (-)
**radiotherapy**^a^			
yes	91 (86.7)	209 (65.1)	300 (70.4)
no	14 (13.3)	112 (34.9)	126 (29.6)
missing	12 (-)	77 (-)	89 (-)
**endocrine therapy**^a^			
yes	0 (0.0)	6 (2.5)	6 (2.0)
no	65 (100.0)	236 (97.5)	301 (98.0)
missing	52 (-)	156 (-)	208 (-)
**immunotherapy**^a^			
yes	2 (3.8)	77 (32.1)	79 (27.1)
no	50 (96.2)	163 (67.9)	213 (72.9)
missing	65 (-)	158 (-)	223 (-)
**bone marrow transplantation**^a^			
yes	0 (0.0)	22 (10.2)	22 (8.2)
no	51 (100.0)	194 (89.8)	245 (91.8)
missing	66 (-)	182 (-)	248 (-)
**earlier tumors known**^a^			
yes	5 (5.0)	26 (7.8)	31 (7.2)
no	95 (95.0)	306 (92.2)	401 (92.8)
missing	17 (-)	66 (-)	83 (-)
**endocrinological co-morbidities**^b^			
none	59 (51.8)	146 (38.8)	205 (41.8)
1	31 (27.2)	134 (35.6)	165 (33.7)
2	20 (17.5)	64 (17.0)	84 (17.1)
3 or more	4 (3.5)	32 (8.5)	36 (7.4)

Data base: ELLY survey (n = 515); absolute (and relative) frequencies. HL = Hodgkin lymphoma; NHL = non-Hodgkin lymphoma; n = number of cases; SD = standard deviation.

^a^ registry data, percentages based upon number without missings.

^b^classification: One or more of the following conditions has been diagnosed: dyslipidemia, hyperthyroidism, hypothyroidism, nodes of the thyroid gland, struma diffusa, diabetes mellitus and osteoporosis?

### Sleep quality

On a range from 0 to 21 the mean self-reported sleep quality was 6.2 (CI 5.9–6.6) (HL 5.8; (CI 5.1–6.5); NHL 6.4 (CI 6.0–6.7)) (Table 2). In total, around half (51.8%) of the survivors were classified as good sleepers (HL 60.7%, NHL 49.0%), with a clear statistically significant distinction between the cancer entities (p = 0.031). The intake of sleeping medication during the previous month (prescribed or “over the counter”) was reported only in a minority of patients (9.7%).

**Table 2:** Sleep quality.

	HL	NHL	total
**PSQI (total score)**	5.8 (5.1–6.5)	6.4 (6.0–6.7)	6.2 (5.9–6.6)
**PSQI subscales**			
subjective sleep quality	1.1 (1.0–1.3)	1.1 (1.1–1.2)	1.1 (1.1–1.2)
sleep latency	1.1 (0.9–1.3)	1.2 (1.1–1.3)	1.2 (1.1–1.3)
sleep duration	0.7 (0.5–0.8)	0.6 (0.6–0.7)	0.6 (0.6–0.7)
habitual sleep efficiency	0.7 (0.5–0.9)	0.9 (0.8–1.0)	0.9 (0.8–1.0)
sleep disturbances	1.1 (1.0–1.3)	1.2 (1.2–1.3)	1.2 (1.2–1.3)
use of sleeping medication	0.1 (0.0–0.2)	0.2 (0.2–0.3)	0.2 (0.2–0.3)
daytime dysfunction	0.9 (0.8–1.1)	1.0 (0.9–1.1)	1.0 (0.9–1.1)
**PSQI (categorized**^a^**)** *absolute and (relative) frequencies*			
good sleepers	68 (60.7)	173 (49.0)	241 (51.8)
poor sleepers	44 (39.3)	180 (51.0)	224 (48.2)
**Chi-Square Pearson** (p-value)	0.031		

Data base: ELLY survey; mean and (confidence interval).

HL = Hodgkin lymphoma; NHL = non-Hodgkin lymphoma; PSQI = Pittsburgh Sleep Quality Index.

^a^total score categorized into good (≤ 5) and poor sleepers (>5).

### SF-36v1

The survivors showed a good overall physical (PCS 44.7; CI 43.6–45.8) and mental (MCS 47.0; CI 45.8–48.3) quality of life (Table 3). The means of all subscales including their CIs were below the norm’s mean (50) but still within one SD (±10), indicating that their self-reported HRQOL was not substantially reduced. Throughout all SF-36 scales, NHL survivors reported lower HRQOL than HL survivors. These differences reached statistical significance with regard to physical functioning and physical role functioning.

**Table 3:** HRQOL, depression, exhaustion and health concept.

	HL		NHL		total	
**SF-36v1**^a^						
physical health component summary score (PCS)	47.1 (44.9–49.4)	[11.7]	44.0 (42.7–45.2)	[11.6]	44.7 (43.6–45.8)	[11.7]
mental health component summary score (MCS)	47.5 (45.2–49.8)	[12.1]	46.9 (45.4–48.4)	[13.7]	47.0 (45.8–48.3)	[13.3]
**SF-36v1 subscales**						
physical functioning	45.8 (43.3–48.3)	[13.7]	41.6 (40.3–42.9)	[13.2]	42.6 (41.4–43.8)	[13.4]
physical role functioning	47.2 (44.9–49.4)	[12.0]	42.0 (40.6–43.4)	[13.8]	43.2 (41.9–44.4)	[13.6]
bodily pain	48.2 (46.1–50.3)	[11.4]	47.8 (46.7–49.0)	[11.3]	47.9 (46.9–48.9)	[11.3]
general health perceptions	47.3 (45.0–49.5)	[12.0]	45.0 (43.9–46.1)	[11.0]	45.5 (44.5–46.5)	[11.3]
vitality	46.7 (44.4–49.0)	[12.6]	44.5 (43.2–45.7)	[12.6]	45.0 (43.9–46.1)	[12.6]
social role functioning	44.5 (42.0–47.1)	[14.0]	43.3 (41.9–44.7)	[14.2]	43.6 (42.4–44.8)	[14.1]
emotional role functioning	46.7 (44.1–49.2)	[13.5]	44.5 (42.9–46.1)	[15.3]	45.0 (43.6–46.3)	[14.9]
mental health	49.9 (48.0–51.9)	[10.6]	48.7 (47.6–49.9)	[11.6]	49.0 (48.0–50.0)	[11.4]
**clinically relevant depression most likely present (WHO-5 wellbeing index)**	*absolute and (relative) frequencies*					
yes	13 (11.3)		40 (10.8)		53 (11.0)	
no	102 (88.7)		329 (89.2)		431 (89.0)	
**Chi-Square Pearson** (p-value)	0.889					
**increased lack of energy, tiredness and inefficiency**^b^						
yes	52 (45.2)		221 (56.5)		273 (54.0)	
no	63 (54.8)		170 (43.5)		233 (46.0)	
**Chi-Square Pearson** (p-value)	0.033					
**health concept** "I am cured"						
yes	98 (86.0)		275 (74.9)		373 (77.5)	
no^c^	16 (14.0)		92 (25.1)		108 (22.5)	
**Chi-Square Pearson** (p-value)	0.014					

Data base: ELLY survey; mean and (confidence interval) and [standard deviation]. HRQOL = health-related quality of life; SF-36 = Short Form Health Survey; HL = Hodgkin lymphoma; NHL = non-Hodgkin lymphoma.

^a^norm-based SF-36 scores for the German population, range: 0–100, with higher values representing higher HRQOL.

^b^response to the question whether after the malignant disease, participants feel an increased lack of energy, tiredness and inefficiency.

^c^because of recurrence, metastases or second cancer diagnosis.

### Clinically relevant depression and health perception

Around 1 in 9 survivors met the criteria of a likely clinically relevant depression according to the WHO-5 (11.0%; n = 53), with similar proportions among the different entities. Additionally, 54.0% of cancer survivors (n = 273) reported an increased lack of energy, tiredness and inefficiency after their malignant disease. NHL survivors were slightly more affected (56.5%; n = 221) than survivors of HL (45.2%, n = 52). 77.5% (n = 373) stated to be cured at the time of the study. NHL survivors less often reported to be cured (74.9%, n = 275) than the HL survivors (86.0%; n = 98). The distribution of exhaustion and experienced cure differed significantly between the entities (p = 0.033 and 0.014, respectively).

### Predictors of sleep quality

The results of the regression analysis with sleep quality as the dependent variable (expressed by the PSQI score) are shown in Table 4. More than a third of variance in sleep quality was explained by the included, pre-specified predictors (R^2^ 0.381; adj. R^2^ 0.358). The type of cancer (entity) did not have a significant influence on sleep quality. Sleep quality was significantly predicted by age beyond 64 years, female gender, not being able to work due to different causes, 3 or more endocrinological co-morbidities, the probable presence of a depression as well as exhaustion (operationalized as an increased lack of energy, tiredness and inefficiency). The latter two variables along with the highest age group (≥ 75 years) exerted the greatest β, corresponding to the most important relative impact on sleep quality.

**Table 4:** Regression analysis with predictors of sleep quality.

	dependent variable: PSQI-Score (0–21)^†^		
	regression coefficient β	p-value	standard error
**age at survey (ref. ≤ 50 years)**			
51–64 years	0.034	0.942	0.462
65–74 years	1.429	0.034*	0.670
≥ 75 years	1.675	0.020*	0.718
**entity (ref. NHL)**			
HL	0.070	0.853	0.378
**sex (ref. male)**			
female	0.779	0.015*	0.319
**marital status (ref. married, civil union)**			
living apart / widowed / divorced / single	0.267	0.472	0.371
**occupational status (ref. education or work**^a^**)**			
not working^b^	1.982	<0.001***	0.514
retirement	-0.887	0.120	0.569
**endocrinological co-morbidites (ref. none)**			
1 co-morbidity	0.185	0.611	0.365
2 co-morbidities	0.248	0.567	0.432
3 or more co-morbidities	1.374	0.031*	0.634
**presence of depression most likely**	3.431	<0.001***	0.517
**“I am not cured” (ref. “I am cured”)**	0.268	0.487	0.385
**exhaustion (ref. not applicable)**			
increased lack of energy / tiredness / inefficiency	2.496	<0.001***	0.322
**R**^**2**^	0.381		
**adjusted R**^**2**^	0.358		

Data base: ELLY survey. PSQI = Pittsburgh Sleep Quality Index.

^**†**^ higher PSQI-scores represent worse sleep quality.

*p<0.05.

***p<0.001.

ref. = reference category.

^a^education/reeducation/studies/housework/fulltime/part-time.

^b^due to unemployment, illness, inability to work, premature pension or disability.

### Sleep quality and health-related quality of life

**Fig 1:**
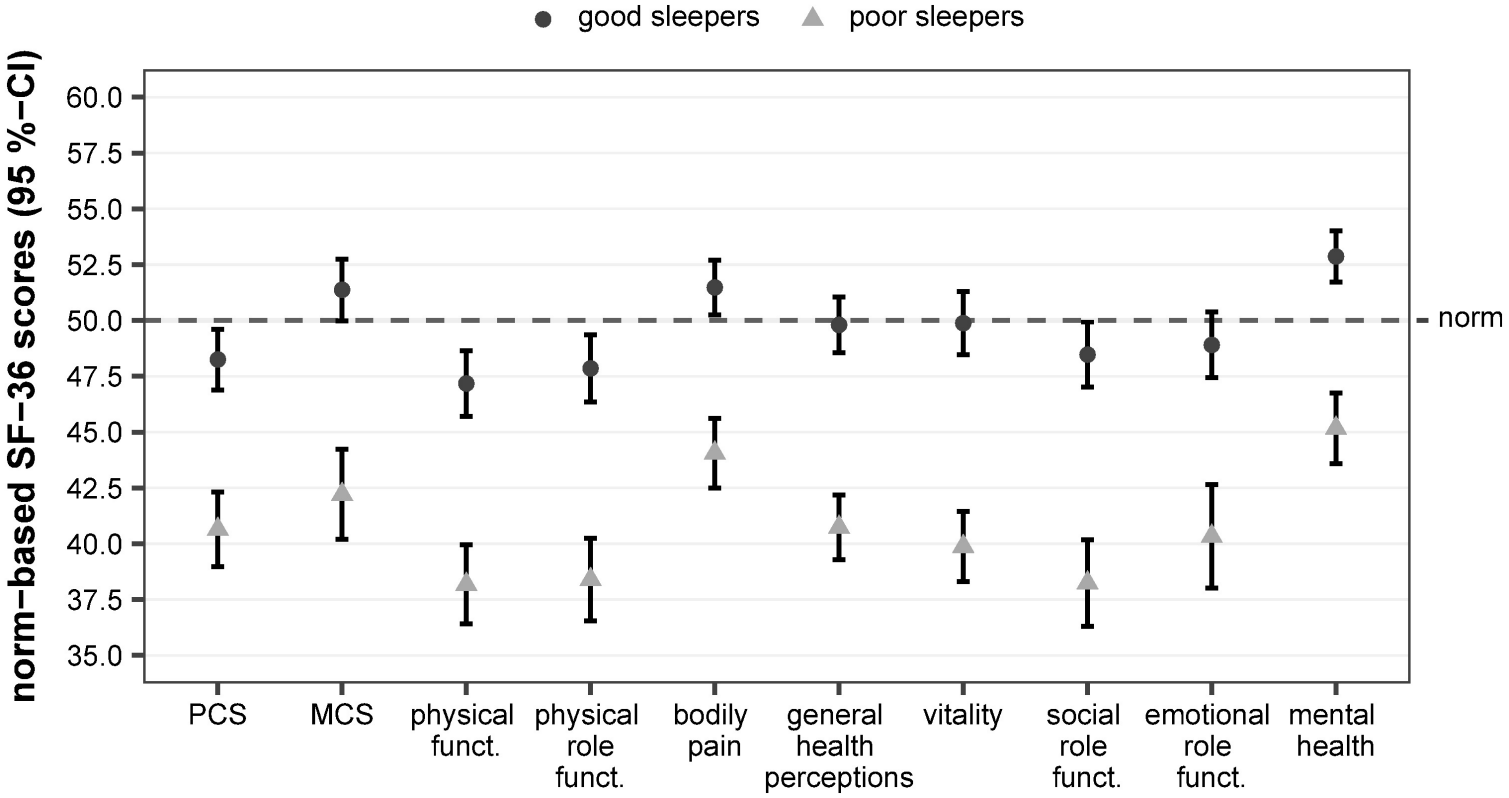
Sleep quality and health-related quality of life in HL and NHL survivors (data base: ELLY survey). Norm-based SF-36 scores for the German population; range: 0–100 with higher values representing better health-related quality of life; PCS = physical health component summary score; MCS = mental health component summary score; funct. = functioning.

Fig 1 shows the HRQOL of the cancer survivors dichotomized into good and poor sleepers, respectively, who differed significantly on all SF-36 scales. In addition to poor sleep, this group also reported a worse HRQOL.

Whereas good sleepers’ HRQOL was within the normal range (50±10(SD)), poor sleepers had an impaired HRQOL (<40) on the subscale physical functioning (38.2; CI 36.4–39.9). Additionally, further scales were indicative of impaired health-related quality of life: physical role functioning (38.4; CI 35.5–40.3), social role functioning (38.2; CI 36.3–40.2), vitality (39.9; 38.3–41.5), emotional role functioning (40.3; CI 38.0–42.6), general health (40.7; CI 39.3–42.2) and PCS (40.7; CI 39.0–42.3).

## Discussion

Along with the increase in survival rates of cancer patients, achieving a good HRQOL of survivors gains importance in the treatment and management of cancer patients [4]. Impairments in HRQOL have been found in HL and NHL survivors [8, 16, 20, 21]. HRQOL can be affected by sleeping problems [7–11], which are a known and common side-effect of cancer [12–15]. This association has been intensively examined for breast cancer patients [7, 10, 12, 14]. As regular follow-ups for cancer patients end after 5 years of recurrence-free survival, fewer data concerning these conditions are available on long term cancer survivors. This study included long-term survivors of HL and NHL 5–15 years after diagnosis. Our findings regarding sleep quality fit in with previous studies on sleep in HL and NHL survivors [8, 13, 16], but also with studies on survivors of other cancer entities [12, 14, 15].

When our study participants were analyzed in total as one group, the reported HRQOL was within the normal range and thus contrasting earlier findings [8, 16, 20, 21]. However, in a subgroup analysis assessing HRQOL according to sleep quality, this result is rather applicable for good sleepers (Table 3). Poor sleep was accompanied by subnormal HRQOL on the majority of subscales of the SF-36 in accordance to some previous studies performed on long term cancer survivors with different cancer entities [7, 9–11]. Our deviations in results regarding the HRQOL of the total population might be due to the fact that earlier studies on HL and NHL survivors mostly referred to distinct hospital patients as participants, with a possibly worse health status than that of survivors recruited via the population-based cancer registry.

The magnitude of the depicted association between sleep quality and HRQOL becomes even more apparent with the fact that 5–15 years after first cancer diagnosis, 51.8% of the HL and NHL survivors reported poor sleep quality. These results demonstrate a high prevalence of sleeping difficulties in the study group compared to representative studies performed on the Austrian and German adult population revealing an overall prevalence of 31.2% and 30.3%, respectively [29, 30].

Our results underpin the hypothesis of a multifactorial etiology of sleeping problems. However, in our study sleep quality of long-term survivors was not significantly influenced by the different cancer entities. Older age constituted a risk factor for poor sleep quality. Although this fact has been previously reported in cancer survivors [18, 19, 31], it remains unclear what proportion of this association can be attributed to the fact that older people in general tend to have poorer sleep quality than younger people [32]. Similar to our result of female gender being a risk factor for poor sleep quality, in other studies female gender was associated with fatigue in HL and NHL survivors [16, 19]. Additionally, endocrinological co-morbidities were an important predictor of sleep. Co-morbidities in general are associated with fatigue in HL as well as NHL survivors [16, 31]. These findings might point to side-effects of drugs’ interactions, taken due to the co-morbidities, which affect sleep. As co-morbidities were also associated with fatigue in the control group of one study, it seems that co-morbidities themselves exert great influence on sleep [16]. The likely presence of depression was associated with the greatest relative impact on sleep quality. The relation between depression and sleep is well-researched with a preponderance of female patients [33], thus it is highly plausible that survivors with depression reported worse sleep quality. However, the prevalence of depression is not elevated in our population when compared to the general population [34], so we can assume that the earlier mentioned excess of poor sleepers is not solely due to a selected study population. The inability to work is associated with poor sleep quality. Beyond the known association between unemployment and depression [35], unemployment might influence sleep in terms of a psychological stressor: Gainful employment implies not only greater chances of participation in social life. Moreover, it facilitates a return to the life before cancer and therefore contributes to one’s self-efficacy. It also improves financial security. Besides these factors, exhaustion after the disease was another predictor of worse sleep quality in our study.

A major strength of the present study is the recruitment from a population-based cancer registry, which resulted in a large, representative study population [22] and a long follow-up time. Another important addition of this study was the inclusion of the social setting and health-related factors, which enabled explaining a high percentage of variance in sleep quality in the regression analysis. With transforming the SF-36v1 scores into norm-based scores, we could make impairments in cancer survivors’ HRQOL visible and comparable to the norm-based scores of the German population. To the authors’ best knowledge, this was the first study which examined an association between sleep and HRQOL in the entities of HL and NHL survivors and yielded dependable data on the prevalence of poor sleepers. There are several limitations to the study. With a response rate of 54.7% some response bias, usually present in survey studies, can be expected: People with chronic health conditions or affected by exhaustion may not be able to participate. However, people with a very good health status may lack motivation to participate in the survey. Furthermore, we did not include any data on treatment in the regression as this information was not recorded with enough detail within the registry and large amount of missing data will not allow sensible subgroup analysis. However, during the study period treatment of NHL was modified with the addition of the monoclonal anti-CD 20 antibody rituximab to the standard chemotherapy cyclophosphamide, doxorubicin, vincristine, and prednisone (CHOP) in 2002 and significantly improved patients’ survival [36, 37]. Additionally, adaptions of radiotherapy treatment with reduced radiation volumes and doses, as well as a decrease in the overall use of consolidative radiotherapy in the treatment of NHL patients in the last two decades has been proposed to reduce therapy-related toxicity [38, 39]. These therapeutic changes may have led to fewer therapy-related late effects such as poor sleep in a proportion of patients in our study group. However, previous studies could not demonstrate an effect of different cancer treatment on sleep quality [19, 31]. Our data were derived from a population-based cancer registry, which does not include details about the localization of radiotherapy. Apart from earlier malignancies, endocrinological co-morbidities, depression and exhaustion, we did not investigate the presence of further health impairments, for example chronic heart failure, chronic kidney diseases or other disabilities, which could also affect sleep quality and HRQOL. The likelihood of clinically relevant depression was examined with the WHO-5 well-being index, which is a screening instrument for depression but does not function as a diagnostic tool. Due to the cross-sectional study design, it is impossible to infer causality. Both HRQOL and sleep quality are part of multifactorial processes. HRQOL incorporates psychiatric aspects, which are highly correlated with sleep. Impaired sleep quality, on the other hand, compromises many areas, e.g. immunological functioning, wound healing and sensation of pain [17], which are again reflected in one’s HRQOL.

In this large study from a population-based regional cancer registry, an association of poor sleep and impaired HRQOL in HL and NHL survivors could be demonstrated. Risk factors explaining more than 30% of the variance in sleep quality, including depression, exhaustion, higher age, inability to work, endocrinological disorders and female gender could be identified. Our results showed that sleeping problems can be present in every second long-term survivor of HL and NHL, and may cause the observed differences in the survivors’ HRQOL. Longitudinal studies are needed to establish a possible causal link as well as to elucidate the impact of further co-variables to develop interventions to mitigate and even prevent psychological strain. Until now, only a few strategies exist for the management and the alleviation of fatigue or sleeping problems in cancer patients and survivors [40]. Based on the findings of the present study, sleep quality should be routinely examined in long-term survivors in order to offer interventions with well-known positive effects on sleep quality such as exercise and psychosocial interventions to affected patients [40].
